# Toxic *Alexandrium* Treatment in Western Australia: Investigating the Efficacy of Modified Nano Clay

**DOI:** 10.3390/toxins17100495

**Published:** 2025-10-04

**Authors:** Cherono Sheilah Kwambai, Houda Ennaceri, Alan J. Lymbery, Damian W. Laird, Jeff Cosgrove, Navid Reza Moheimani

**Affiliations:** 1 Algae Innovation Hub, Murdoch University, Murdoch, WA 6150, Australia; sheilah.kwambai@murdoch.edu.au (C.S.K.); d.laird@murdoch.edu.au (D.W.L.);; 2 Centre for Sustainable Aquatic Ecosystems, Harry Butler Institute, Murdoch University, Perth, WA 6150, Australia; 3 Department of Biodiversity, Conservation and Attractions, 17 Dick Perry Ave, Kensington, WA 6151, Australia

**Keywords:** harmful algal blooms, *Alexandrium* spp., modified nano-clays, kaolinite, socio-economic impacts

## Abstract

*Alexandrium* spp. blooms produce a range of toxins, including spirolides, goniodomins, and paralytic shellfish toxins (PSTs). Of these, PSTs are the most impactful due to their high affinity for voltage-gated sodium ion channels in nerve cell membranes. This interaction can cause neurological effects such as paralysis and, in severe cases, may lead to death. Given the implications of *Alexandrium* blooms on public health, all mitigation, prevention, and treatment strategies aim to reduce their socioeconomic impacts. However, monitoring harmful algal blooms remains difficult due to confounding influences such as pollution, climate change, and the inherent variability of environmental conditions. These factors can complicate early detection and management efforts, especially as the intensity and frequency of blooms continue to rise, further exacerbating their socioeconomic consequences. This review offers insights into several management approaches to prevent and control *Alexandrium* blooms, focusing on modified nano-clays as a promising emergency mitigation measure for low-density toxic algal blooms, especially in areas predominantly used for recreational fishing. However, it is recommended that treatment be coupled with monitoring to alleviate reliance on treatment alone.

## 1. Introduction

**Figure 1:**
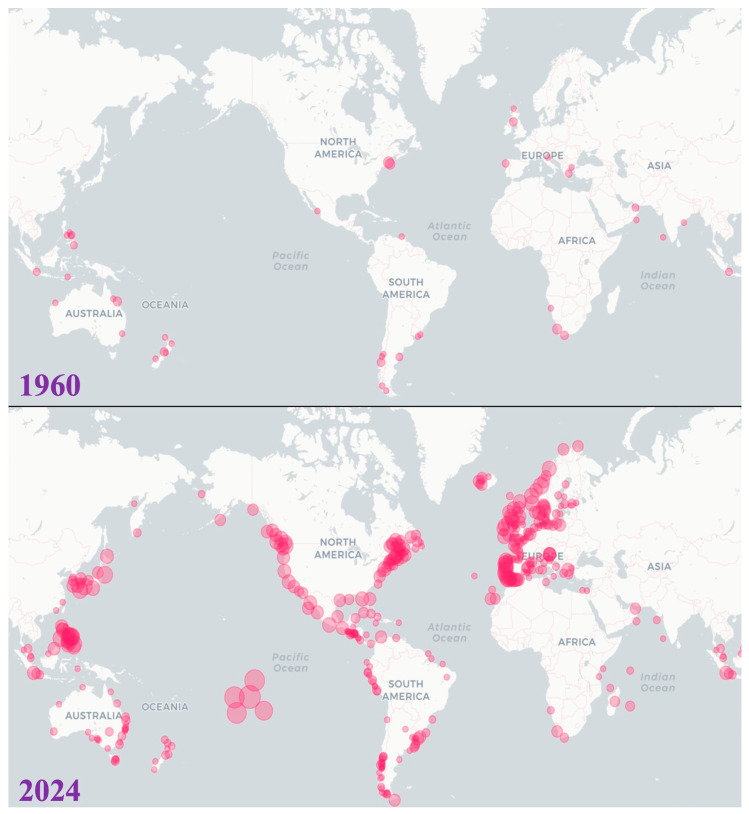
Occurrences of harmful algal blooms and their impacts on various ecosystem components, including humans (all poisoning syndromes), aquaculture, shellfish, aquatic animals, fish, and birds (figure made with data, https://data.hais.ioc-unesco.org/ 27 February 2025). Circle size corresponds to the number of harmful algal event records (or aggregated count/intensity) in that location/time window.

The frequency and intensity of harmful algal blooms and their associated human poisoning syndromes have increased over the past half century (Figure 1) [1]. Roughly 30% of harmful algal bloom (HAB) species produce natural toxins that can cause harm to the environment [2,3,4]. These “marine biotoxins” are produced via secondary metabolic pathways, primarily in dinoflagellate and diatom HAB species. [5]. Marine biotoxins embody a diverse array of chemical compounds associated with various syndromes that affect humans, fish, reptiles, marine mammals, cephalopods, and seabirds [5]. When transferred through the food web, these biotoxins can produce a range of effects in marine organisms, including slowed growth, deformities, altered behaviour, and death [6].

Toxic HAB outbreaks result in detrimental ecological effects and have economic ramifications on commercial and recreational fisheries, aquaculture, and tourism [7]. Consequently, these blooms can lead to significant socio-economic impacts [1].

Species from the dinoflagellate genus *Alexandrium* have a wide geographical distribution, are resilient, and can survive in variable marine habitats and hydrographic regimes [8,9], making bloom outbreaks of particular interest for management and mitigation. This resilience is mainly attributed to their ability to form cysts, both temporary and resting [10]. *Alexandrium* spp. form temporary (pellicle) cysts as a sensitive response to adverse environmental conditions, including unfavourable temperatures, intense light, high salinity, and nutrient deficiency, allowing re-germination for up to 17 days post cyst formation [10,11,12,13]. Resting cysts are a longer-term survival mechanism, forming when growth conditions deteriorate and remaining viable for timescales ranging from a month to several years, serving as survival ‘pool’ that can be reactivated when optimal conditions for growth return [14].

At least 50% of the 30+ morphologically defined species of *Alexandrium* are known to be toxic or have harmful effects, with PSTs accounting for a significant portion of those harmful effects [15]. Paralytic shellfish toxins are naturally occurring neurotoxic alkaloids that mainly pass through the marine food web via vector organisms such as filter-feeding invertebrates, although there are reports of gastropods and planktivorous fish serving this role [16,17]. Saxitoxin, the parent compound in the PST suite, is the most studied PST compound since its discovery in clams in 1957 [16]. To date, 57 analogues have been described, with each having different toxic capabilities [18,19]. Paralytic shellfish toxins can cause paralytic shellfish poisoning (PSP), a severe and occasionally fatal illness resulting from the consumption of PST-contaminated seafood [20,21,22,23].

*Alexandrium* is a common HAB genus in Australian waters [24] with eleven species of *Alexandrium* currently described, four of which are PST-producing [25,26]. Research on *Alexandrium* in Australia has been focused primarily on the waters of the south-eastern region of the continent, with particular attention paid to changes in population distribution and the threat to aquaculture [27,28,29,30].

**Figure 2:**
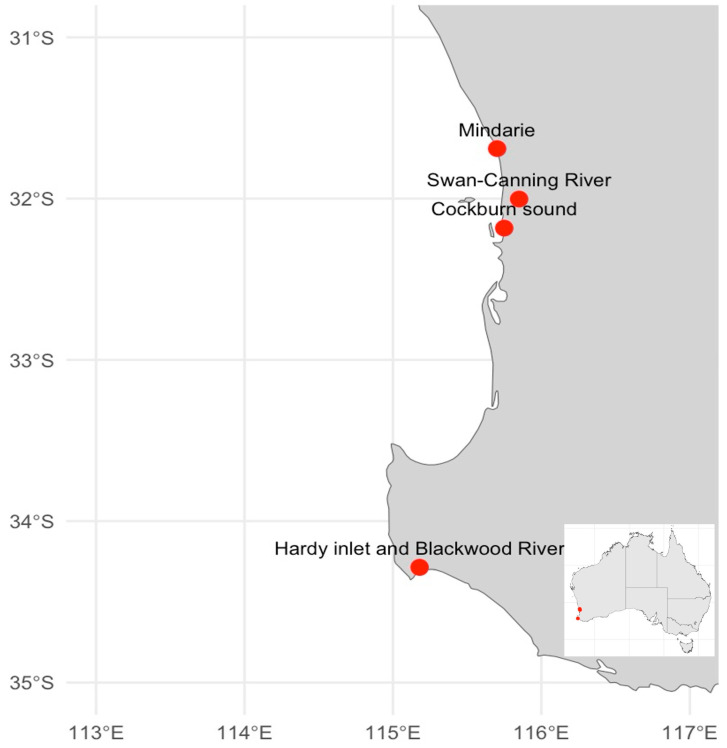
Map showing reported locations of *Alexandrium* spp. in the Swan Canning Estuary, Mindarie, Cockburn Sound and Blackwood River in Western Australian Coast.

In Western Australia (WA) blooms of two species of *Alexandrium* have been recorded. *Alexandrium minutum* was first reported in the Swan Canning Estuary, which flows through the city of Perth, in 1983 [31]. Since then, this species has also been reported about 300 km south of Perth, in the Hardy Inlet [31]. It is likely that *A. minutum* was transported by the Leeuwin Current, which drives the southward dispersal of marine organisms in the state (see Figure 2) [32]. Blooms of *A. pacificum* were first confirmed in Cockburn Sound, and this species that has also been regularly reported in water samples from Mindarie Marina since 2018 [33,34]. Both Cockburn Sound and Mindarie are coastal waters adjacent to the Perth metropolitan area. The first major bloom of *Alexandrium* spp. that resulted in a large-scale response from management authorities occurred in the Swan-Canning River during early autumn 2019 and re-emerged through the austral summer/autumn of 2019–2020. During these bloom events, toxin levels exceeded the regulatory limit of 800 µg STX di-HCl eq/kg in two important recreational and commercial seafood sources: blue-swimmer crabs (*Portunus armatus*) and the Mediterranean mussel (*Mytilus galloprovincialis*) [35].

Considering the significance of these occurrences, evaluating the potential impacts on commercial and recreational wild capture fisheries, tourism, and broader cultural and social consequences in Western Australia (WA) is crucial. Recent data published by the Department of Fisheries reveal that the total value of commercial fisheries production in WA is estimated at AUD$ 523 million, with aquaculture contributing AUD$ 96 million [36]. The direct and indirect effects of HAB blooms such as *Alexandrium* pose a significant risk to the productivity of aquaculture operations [36]. Uncertainties around the risk posed by local *Alexandrium* species to current and potential future mariculture ventures could limit the growth of this industry.

Various mitigation strategies have been proposed for managing *Alexandrium* blooms and their cysts [13,37,38,39,40,41,42]. This review focuses on the potential of modified nano clays as a solution for addressing toxic *Alexandrium* spp. blooms. It highlights the key mechanisms of action and the advantages of nano clay treatment that have seen it gain favour as a suitable and effective HAB control technique. Further, the review will outline factors that can limit treatment success if not considered and managed appropriately. By thoroughly exploring these aspects, the findings of this review should serve as a solid foundation for coastal and waterway managers considering local HAB management options and provide a basis for resource allocation and management strategies that support the preservation of aquatic ecosystems and socio-economic interests of impacted regions.

## 2. Socio-Economic Impacts of *Alexandrium* Blooms

The socio-economic impacts of *Alexandrium* spp. blooms and other HABs can include economic losses, monitoring and management cost, ecosystem disruption, effects on human health and well-being and social dislocation [43,44]. In essence, during *Alexandrium* spp. blooms, the restrictions and closures imposed on shellfisheries, aquaculture, and recreational areas to protect human health often result in economic and social consequences. Typically, economic impacts are discussed in relation to losses affecting commercial shellfisheries and aquaculture farms. In these industries, impacts can be relatively easily assessed using a market value approach, where losses are linked to changes in availability of goods, services and product recall [5,43]. For example, in the Massachusetts, USA, an *Alexandrium fundyense* bloom event in 2005 had direct economic impacts of $16–18 million while a bloom event that impacted on the US West Coast the razor-clam fishery, reduced tourist numbers to remote areas of Washington State so significantly that up to 339 full-time jobs were lost [1,45]. This Massachusetts bloom event led to increased prices at the Fulton fish market in New York [45] due to product scarcity. These economic effects of fisheries closures also then raise the costs associated with managing and monitoring the fisheries [46]. The recreational impacts of HABs are harder to quantify, as they often rely on non-market value estimates of goods and services [43]. In some instances, recreational areas support local tourism industries; in other cases, cultural resources affect livelihoods and cultural amenities, potentially resulting in long-term consequences for community well-being [47,48]. Closures can disrupt traditional ways of life, impacting sense of place and identity of both individuals and communities: important impacts with long lived consequences that are often overlooked and difficult to quantify [49].

Public health risks associated with blooms of *Alexandrium* spp., primarily due to the production of paralytic shellfish toxins (PSTs), also have significant socioeconomic consequences. These toxins, which target voltage-gated sodium channels in humans, can cause severe neurological effects and even death [18]. Beyond the immediate health implications, such events can lead to widespread economic disruption. For instance, in the Bay of Plenty, New Zealand, at least 20 people were hospitalized in 2012 after consuming contaminated clams (*Paphies subtriangulata*) collected by recreational fishers during an *Alexandrium catenella* bloom event, creating a significant burden on healthcare systems and emergency services [50]. Moreover, such outbreaks often necessitate costly public health interventions, reduce consumer confidence in seafood markets generally, and result in temporary closures of fisheries [46]. These closures not only affect commercial fishers and seafood vendors but also impact recreational fishing and tourism, leading to lost income and increased monitoring and management costs [46].

In addition to effects on human activity and health, *Alexandrium* spp. can have various impacts on ecosystems. For instance, in a coastal lagoon in the Azores Archipelago, located in the North-eastern Atlantic Ocean, *Alexandrium minutum* caused the death of small pelagic fish and resulted in the PST toxification of clams, exceeding 30 times the maximum regulatory limit [51]. In a separate incident in Sagadahoc Bay, Maine, USA, an *Alexandrium* bloom event led to the death of the endangered sturgeon, *Acipenser brevirostrum*, as a result of trophic transfer of PSTs, posing a threat to this vulnerable fish population [52]. Deaths in endangered diamondback terrapins (*Malaclemys terrapin*) due to *Alexandrium fundyense* blooms have also been reported [53].

The identification of HABs generally increase monitoring and management costs [48,54] and monitoring of HABs can be challenging due to the large geographic areas involved, often covering extensive coastal regions, and the complex interactions among environmental processes that contribute to bloom development and persistence. For some HABs, including those caused by species of *Alexandrium*, significant impacts can occur even at low biomass levels increasing the complexity of how to monitor and when to trigger a response. These factors generally necessitate a well-considered, multifaceted monitoring approach [43,54].

## 3. Management of Harmful Algal Blooms

**Figure 3:**
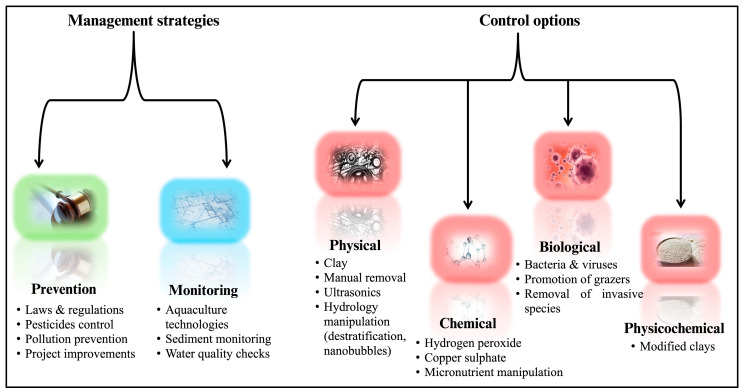
Management strategies and control options for HABs. Red control options icons are considered for emergency-use only and are not recommended for regular application due to environmental and/or economic concerns.

Harmful algal blooms are often complex to manage due to the diffuse sources that contribute to blooms, species diversity, and the variable relationship between biomass and toxicity [15,55,56]. While there is consensus that prevention is the preferred management strategy, implementing this can be challenging [55]. For instance, effective prevention measures require multi-scale studies of specific HAB species involving ecologists, climate scientists, policymakers, and management authorities to understand and prepare for bloom events [57]. Given the reported increase in HAB intensity and frequency [58], there is a growing field of research focused on methods to prevent and control/treat HABs [58,59,60,61]. These methods can be classified into management strategies—such as regulation and project improvements—and control options, where treatment is broadly categorised into biological, physical, or chemical approaches (see Figure 3) [62,63].

### 3.1. Prevention

Ultimately, reducing the occurrence of HABs is the primary objective of most prevention strategies [64]. These strategies may involve nutrient load reduction methods, such as preventing fertiliser overload in agricultural soils, reducing sewage runoff, and implementing groundwater control solutions like installing biofiltration systems and denitrification barriers [43,65,66]. Other strategies include raising awareness of the impacts of increased nutrient loads and HABs and developing supportive policies and guidelines [67]. However, challenges persist, including the increase in nutrient inputs from growing populations and the rise in fossil fuel use and chemical fertilisers [68]. Furthermore, HAB occurrences happen in some areas despite little to no anthropogenic nutrient inputs [59]. Consequently, most policymakers have shifted their focus toward adapting to and mitigating these blooms through monitoring, and in some cases, implementing mitigation strategies prior to bloom formation [7,60,69].

Adaptation measures can also incorporate mitigation actions aimed at reducing the impacts of HABs on ecosystems, human health, and the economy [43]. These can include cultivating resistant species or those that accumulate fewer HAB toxins in aquaculture farms and shellfisheries [70]. In some cases, it has been suggested that using floating covers in small-scale aquaculture farms can prevent HAB growth [63]. Preventing the introduction of HAB species to new areas via ship ballast water is a common measure [27]. For instance, there are reports that hydrogen peroxide (H_2_O_2_) treatment inhibits the germination of *Scrippsiella trochoidea* in ballast water [71].

Monitoring HABs has become one of the most important investments in minimising impact with primary focus on forecasting and predictions. For instance, ocean colour imagery from the Sea-viewing Wide Field-of-View Sensor (SeaWiFS) aboard the OrbView-2 satellite has been used to track and monitor red tide blooms in Florida in near real-time [72]. However, the high costs associated with this technology are compounded by limitations such as interference from cloud cover and the restricted resolution for distinguishing different phytoplankton communities [55,73]. Other approaches to monitoring include the use of cell count data and image-based techniques (e.g., light microscopy). These methods can be time-consuming and may be ineffective in accurately identifying phytoplankton species, which presents a bottleneck, especially when identifying cryptic *Alexandrium* spp. that have nearly identical morphological features but differ genetically [74,75]. Some *Alexandrium* species, such as *Alexandrium minutum*, can also exist as toxic and non-toxic variants [74].

This could potentially safeguard aquaculture operations, protect public health, and preserve the cultural and recreational value of affected waterways. Integrating early warning systems, such as real-time PCR for species-specific identification and HAB alarm systems, into routine monitoring programs could improve early bloom detection [76]. These tools may enhance management strategies, enable timely deployment of modified clay treatments, and improve the cost-effectiveness of bloom mitigation. In turn, this could help protect aquaculture, public health, and the cultural and recreational value of impacted waterways.

### 3.2. Treatment

Treatment of HABs can include, but is not limited to, manual removal, application of copper sulphate or algicidal bacteria, micronutrient manipulation, and flocculation and sedimentation [77,78,79]. These approaches are broadly classified into physical, biological, and chemical control categories [80,81], and are primarily implemented as emergency countermeasures to suppress large-scale blooms [80]. Physical methods such as skimming, disruption, and isolation can effectively eliminate HAB cells from the water column [62]. These techniques are generally straightforward and cause minimal secondary impacts, making them suitable for targeted or small-scale applications, although large-scale deployment may be limited by cost considerations [62,81]. Table 1 provides a summary of the effectiveness, scalability, and environmental trade-offs associated with various HAB control methods.

Biological treatment methods involve algicidal bacteria, viruses, protistan grazers and niche competitors [60,63,80]. Several studies have investigated the effectiveness of these treatment methods. For instance, the protistan grazer ciliate *Strombidinopsis jeokjo* reduced large populations of *Margalefidinium polykrikoides* [82]. In another example, algicidal bacterium *Altererythrobacter* sp. was reported to control HABs caused by *Alexandrium tamarense* [83]. However, there are disadvantages to using algicidal bacteria. Firstly, these methods require the isolation of specific strains [83] and there are concerns about using one organism to control another because released organisms can become invasive species and may have unintended environmental impacts (see Table 1). As a consequence, biological control in most regions requires extensive permits limiting its use [81]. Finally, allelochemicals have been used in some cases to inhibit the microalgal overgrowth of HABs [84]. While this approach can promote biological competition and is considered environmentally friendly [84], it has drawbacks. For example, different *Alexandrium* species and strains vary in their PSP toxin content and composition, and these toxin profiles respond differently to the presence of allelochemicals, regardless of cell survival [8].

Chemical methods utilise toxic substances to control HABs, such as hydrogen peroxide, sodium hypochlorite, chlorine, cupric sulphate, and sophorolipid [85,86,87]. One of the first chemical treatments used copper sulphate (CuSO_4_) to suppress *Karenia brevis* blooms [88]. However, while CuSO_4_ initially suppressed the blooms, it did not completely terminate them, leading to recurrence and causing harm to the marine ecosystem [62,63]. In a separate instance, hydrogen peroxide (H_2_O_2_) successfully reduced the concentrations of vegetative cells and pellicle cysts of toxic *Alexandrium ostenfeldii* by up to 99.8% during a bloom event [38]. H_2_O_2_ can degrade into water and oxygen within a few days, allowing the ecosystem to recover quickly after treatment [38]. However, other reports have found that H_2_O_2_ can cause unusual swimming behaviour in fish and, in some cases, mortality [89]. Collectively, these findings indicate that the use of non-specific chemical treatments must be carefully considered, with applications tailored and dosed according to the local biogeochemical environment. Moreover, applying chemicals to control large-scale blooms would be operationally difficult and cost prohibitive.

**Table 1:** Summary of removal technique, effectiveness, scalability and environmental trade-offs of various HAB control methods.

Category	Method	Removal Technique	Effectiveness & Scalability	Environmental Trade-Offs
Biological	Algicidal bacteria	Species-specific targeting; Cell destruction via enzymes [90].	Time-consuming to isolate; Requires high-yield production; Expensive.	May affect non-target species; Environmental risks from biological release [91].
	Algicidal viruses	Infect and lyse algal cells [80].	Naturally abundant; High host specificity; Easy to apply [80].	Limited practical application; Potential non-target effects [91].
	Allelochemicals	Inhibit or alter algal growth and reproduction [92,93].	Low cost; Limited field data; Biodegradable; Variable effectiveness [80,84].	May affect biodiversity; Non-targeted species impacts [94].
	Protozoan grazers	Predate and feed on HABs can be species-specific [95].	Experimental; Potential for large-scale use.	Non-specific feeding; Risk of trophic imbalance; May increase toxicity [63].
Chemical	Algicides (i.e., H_2_O_2_, CUSO_4_)	Rapid oxidative damage to algal cells and cysts [63].	Effective for large-scale use; Short-lived effects; Risk of bloom recurrence [38].	Fish behavioural changes; Oxygen depletion; Human health and water risks [62,63,89,95].
	Engineered Nanoparticles (e.g., TiO_2_, silver, magnetic NPs)	Adsorption via electrostatic interaction [96].	Expensive; Limited scalability [80,95].	Potential toxicity to environment and organisms [95].
Physical	Ultraviolet radiation	Damages algal metabolism and cell integrity [80].	Not suitable for large-scale use; Equipment and manpower intensive effectiveness; light penetration limitations [95,97]. Not suitable for large-scale use; Equipment and manpower intensive effectiveness; Light penetration limitations [95,97].	May disrupt aquatic organisms and microbial community; toxin release trade-off [95,97]. May disrupt aquatic organisms and microbial community; toxin Toxin release trade-off [95,97].
	Ultrasonication	Cell lysis and photosynthesis inhibition [98].	High removal efficiency; Not scalable; High energy demand [80].	Water quality impacts; Harm to non-target organisms; Toxin release risk [80,99,100].
Physicochemical	Modified clays	Flocculation and sedimentation of algal cells [81].	Scalable; Low cost; Low energy demand high removal efficiency [13,101].	Low doses minimal to no environmental impacts; Potential long term impacts on benthic and non-target aquatic organisms [81,101].
	Sophorolipid (Biosurfactant)	Disrupt algal cell membranes [102].	Large scale applicability; selective action; high cost; low toxicity at low dosage [102]. Large scale applicability; selective action; High cost; Low toxicity at low dosage [102].	Potential non-target effects; Industrial-scale development still under development [102].

Finally, the most reliable and convenient method for large-scale HAB management is the combined use of physical and chemical approaches [62,76,81]. These physico-chemical methods typically involve natural minerals, such as clay, to remove HAB cells through flocculation and sedimentation [60,62,63]. However, natural clay has low coagulation efficiency, requiring high clay loads that may lead to substantial deposition and potential disruption of the benthic environment [80]. To address this, researchers have explored chemically augmented clays as a promising alternative. Augmentation techniques often involve the addition of inorganic and organic compounds—commonly used in water treatment for decades, such as polyaluminum chloride and aluminium sulphate [76,81]. These modified clays (MC) have been successfully implemented in China [81] and Korea [76]. The additives enhance the clay’s surface area and positive charge, increasing its affinity for algae and improving flocculation efficiency [81,103,104,105]. Importantly, MCs must be tailored to specific target species and water chemistry, as no single formulation is universally effective across all bloom types or aquatic environments.

## 4. Treatment of HABs Using Nano Clays

Nano clay refers to clay minerals that have been reduced to the nanoscale, typically less than 100 nm in at least one dimension, which gives them a much higher surface area and reactivity compared with conventional clay. The use of nano clays to treat harmful algal blooms (HABs) dates back to 1989 [106]. The fundamental principle behind treating HABs with clay is flocculation—interactions between algal cells and clay particles lead to the formation of larger flocs that settle to the bottom of the water body [107,108]. The larger surface area of nano clays enhances their flocculant ability, promoting more effective aggregation in algal-infested waters [109,110]. Flocs are formed when HAB species bind to nano clay particles, causing them to clump together and sink as larger aggregates [110]. This process is driven by physico-chemical interactions that induce algal cell mortality [56], resulting in the entrainment of cells among clay particles and their eventual sedimentation [111]. Treatment effectiveness varies greatly between different clay types due to differences in their physico-chemical properties, algal species, dispersal methods, and the hydrodynamic conditions of the specific marine ecosystem being treated. Effective removal of algal cells using clay involves a sequence of steps: initial encounters between cells, ballast, biopolymers, or floccules; subsequent physicochemical interactions such as cation bridging and acid-base attraction; and final sedimentation driven by flocculation or ballast [107,112,113].

### 4.1. How Clay Works and Factors That Influence Binding Efficiency

Treatment of harmful algal blooms (HABs) using clay has been trialed at large scales in several countries, including Japan [106], China [109,114], South Korea [115], and Australia [116]. However, the success of nano clay applications has been variable, likely due to insufficient consideration of the many factors that influence removal efficiency. Among these, flow conditions play a critical role [117], as they directly affect flocculation, deposition, advection, and resuspension behaviour of clay particles [56]. For example, currents and wave action can resuspend clay flocs, inhibiting effective deposition [56]. While turbulence influences particle encounters and disruption, the surface characteristics of the particles determine how strongly they bind to or repel each other. Due to cellular excretion and adsorption, the surface layer of algal cells consists of amino acids, carbohydrates, and organic acids, forming a viscous coating that can ionize or hydrate under seawater conditions, thereby acquiring charge [76]. Different HAB species have unique surface layer compositions, which influence their interactions with clay particles, including the process of coagulation.

The use of coagulants in combination with clay can significantly increase the removal efficiency of harmful algal blooms (HABs), offering a potential win-win scenario. Coagulants enhance the affinity between clay particles and algal cells, thereby improving flocculation and sedimentation [107]. This approach also reduces the amount of clay required for treatment, minimizing clay loadings in the water column and potential environmental impacts [107]. The efficiency of clay-based removal is influenced by several factors, including the type of modifier used, the clay’s chemical composition, surface charge, and surface area. Various modified nano clay treatments have been applied to different algal species under varying conditions, (e.g., clay concentration, pH, temperature, and suspension medium (seawater or deionised water)), to assess their effectiveness (see Table 2).

**Figure 4:**
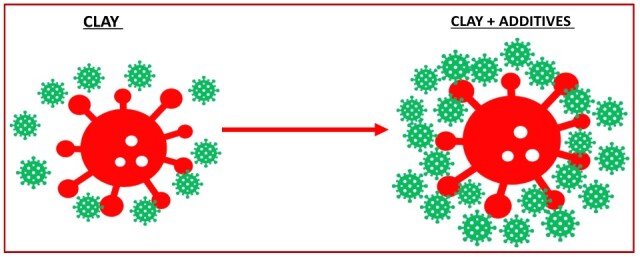
Interactions between clay particles (red) and *Alexandrium* cell (green) before and after addition of additives-modification (polymers, organic compounds, inorganic coagulants).

One of the most critical factors influencing flocculation efficiency is the surface properties of clay particles; therefore, improving harmful algal bloom (HAB) treatment relies on modifying the clay surface [80,81,111,124]. This generally involves increasing the positive surface charge of clay particles and strengthening the bridging interactions between clays and algal cells, which forms the basis of the Clay Surface Modification Theory (CSMT) [110,125,126]. The CSMT incorporates mechanisms that enhance flocculation efficiency by employing modified clays (MC) with higher removal performance [110,125]. Flocculation is essentially a process of contact and adhesion between clay and algal particles. However, particle contact alone does not necessarily result in adhesion; effective collisions are required, and these strongly influence removal efficiency, which is highly dependent on clay surface properties [81,127]. Targeted surface modification of clays improves enmeshment, bridging, and electrostatic interactions, thereby promoting effective collisions and substantially increasing removal efficiency (see Figure 4) [81,125].

Clay surfaces can be modified by using additives such as polymers, poly aluminium chloride [104], inorganic coagulants (e.g., aluminium sulphate, aluminium chloride) [118], and organic compounds; and surfactants such as betaine [42]. These additives enhance clay’s surface area and charge, increasing its affinity for algae [125]. For instance, inorganic coagulants improve flocculation by facilitating cell bridging and enmeshment [81], while polymers such as PAC hydrolyze into positively charged compounds that coat clay particles, reversing their surface charge and promoting adhesion to algae [81,111], including through van der Waals forces. An increase in coagulant concentration decreases the electronegative potential of the clay surface [85].

Consequently, clay modification can increase removal efficiency by several dozen times compared to natural clay [85]. Modified clays have also demonstrated the potential to detoxify PSTs in toxic *Alexandrium* spp. [112]. Therefore, modified nano clays can be used to treat *Alexandrium* spp. and limit socio-economic impacts.

### 4.2. Nano Clays and Alexandrium spp.

Over the years, local governments, researchers, and scientists have made concerted efforts to treat *Alexandrium* blooms and alleviate their impacts through chemical, physical, and biological approaches [60]. Several mitigation methods have been investigated, with one of the most promising being a physico-chemical strategy—specifically, the use of modified clay (MC) [80]. Modified clay produces cationic hydrolysis products that inhibit the growth of harmful algal blooms (HABs), and this approach has been extensively used throughout Southeast Asia [81]. In some cases, MC can also inhibit the germination of cysts [37,119]. Additionally, MC can induce cyst formation in algal cells by creating environmental stress through reduced light intensity, nutrient absorption, and altered oxygen levels—factors that collectively trigger temporary cyst formation [102,119]. These effects suggest that MC can effectively limit algal proliferation and help control HABs.

However, modified clay and other treatment methods have only been tested in a laboratory setting [60,80]. While the laboratory trials presented in Table 3 demonstrate that *Alexandrium* blooms can be effectively treated, the scalability of these treatments is limited in large-scale field applications, particularly when blooms extend over hundreds of square kilometres and there is a heightened risk of adverse secondary impacts on other ecosystem components [60,80].

### 4.3. The Differential Removal of Alexandrium spp. by Aggregation of Nanoclays

The principle behind treating HABs with clay involves the mutual aggregation of algal cells and the clay mineral particles [111]. The clay/algal flocculation is influenced by factors such as algal species size, mineral type, ionic strength of medium, and concentration of both the clay and algal particle [111]. Therefore, factors such as chemical composition, surface charge and surface area, density, size, and shape contribute to the movement and collision between algal cells and clay particles and interparticle forces that affect flocculation efficiency [76,108,111,130]. Aggregation is primarily divided into two sequential steps: transport and attachment.

The main transport mechanisms are Brownian diffusion, shearing, and differential sedimentation, with the effectiveness of each mechanism tied to particle size [111]. The attachment between clay and algal particles primarily depends on the chemical and surface properties of the medium, such as pH, ionic strength, ions valence and polyelectrolytes [81,111]. The exchange of ions with different valences, specific adsorption of charged molecules, and ion exchange collectively aid in developing the surface charge for the clays [111]. Oppositely charged ions balance surface charges on a medium, creating an electrical double layer. Consequently, interactions between particles with similarly charged double layers result in a low electrostatic repulsion and low inclination for aggregation. However, an increase in the concentration of the counterions decreases the thickness of the electrical double layer, thereby reducing repulsion and allowing attractive forces (e.g., van-der-Waals forces) to promote rapid particle aggregation [111,131].

Several factors are known to influence the surface charge of particles, including pH, the ionic strength of the aqueous medium, microalgal species involved, and their growth conditions [131,132,133,134,135,136,137,138]. In general, the flocculation process involves three main interaction mechanisms between algal cells and clay particles: charge neutralization, bridging and attachment [118,139]. Clay treatment has emerged as one of the most attractive methods for managing HABs owing to its low cost and suitability for large-scale applications compared to biological, chemical, or other physical approaches [56,62,76,111,140].

The selection of appropriate clay mineral for treatment of harmful algal species such as *Alexandrium* spp. is a crucial aspect of the mitigation treatment process, as a removal efficiency depends strongly on clay type and structural properties [81,124]. Previous studies have shown that kaolinite clay, in particular, exhibits high removal efficiency in the treatment of HABs [81,110,111,125]. Kaolinite clay is also abundant and mined in several countries due to its commercial value in the construction, ceramic, pottery, and cosmetic industries. We can predict the interactions of kaolinite with *Alexandrium* spp. by using the Derjaguin-Landau-Verwey-Overbeek (DLVO) theory [141].

### 4.4. Kaolinite-Alexandrium Adhesion Mechanism

**Figure 5:**
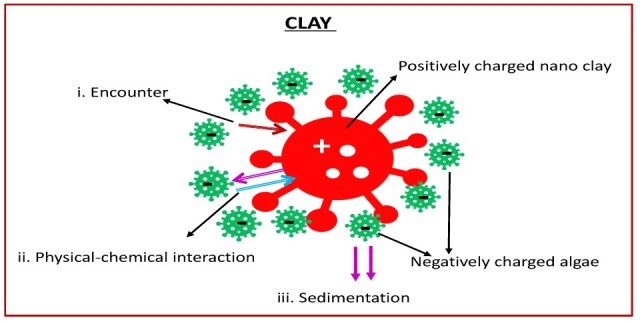
Interactions between clay particles (red) and *Alexandrium* cells (green).

The extended Derjaguin-Landau-Verwey-Overbeek (XDLVO) theory helps to get a better understanding of the interaction between the added nano clays and the microalgal cells (see Figure 5) and enables calculation of the total interaction energy between the clay particles and the *Alexandrium* microalgal cells [141]. According to the XDLVO theory [141], the total interaction energy ($G_{Tot}$) is the sum of electrostatic interactions ($G_{EL}$), Lifshitz-van der Waals interactions ($G_{LVW}$), and acid-base interactions ($G_{AB}$) (Equation (1)) [142]. Each component of these can be either attractive or repulsive depending on the surface properties of the nano clays and *Alexandrium* cells in aquatic environment.

(1) $$G_{Tot}=G_{LVW}+G_{EL}+G_{AB}$$

where *G_LVW_* and *G_EL_* are the Lifshitz–van der Waals and electrostatic interaction energies, respectively. Lifshitz–van der Waals interactions (LVW) are attractive forces originating from instantaneous asymmetrical electron distribution in molecules (apolar component) [143]. Electrostatic forces (EL), resulting from Coulombic interactions between surfaces, are typically repulsive because both the microalgal cell and the substrate carry negative charges. Acid-base forces (AB), on the other hand, arise from electron transfer between the polar components of the microalgal cell and the substrate surface. By thermodynamic convention, a negative value of the total interaction energy (*G_Tot_*) indicates an attractive interaction (adhesion), while a positive value suggests repulsion between the surfaces.

The Lifshitz-van der Waals component of free energy ($G_{LVW}$) between a spherical particle and a semi-infinite plate is calculated as follows [142]:

(2) $$G_{LVW}=\;-\frac{A}{6}\left[\frac{r}{d}+\frac{r}{d+2r}+\ln\frac{d}{d+2r}\right]$$

where *r* is the radius of the spherical particle (m), *d* is the separation distance (m), *A* is the Hamaker constant (Equation (3)), *d*_0_ the minimum separation distance between two surfaces (*d*_0_ = 1.57 × 10^−10^ m), and ${∆G}_{adhesion}^{LVW}$ is calculated from Equation (4) [142]:

(3) $$A=\;-12\pi d_{0}^{2}{∆G}_{adhesion}^{LVW}$$

(4) $${∆G}_{adhesion}^{LVW}=-2\left(\sqrt{\gamma_{c}^{LVW}}-\sqrt{\gamma_{L}^{LVW}}\right)\left(\sqrt{\gamma_{cl}^{LVW}}-\sqrt{\gamma_{L}^{LVW}}\right)$$

where $\gamma^{LVW}$ denotes the Lifshitz-van der Waals component of surface free energy, and the subscripts *c*, *cl*, and *L* refer to the microalgal cell, clay particle, and the water medium, respectively. On the other hand, the Lifshitz-van der Waals interaction between two interacting spheres with radii *r*_1_ and *r*_2_ is calculated using Equation (5), where *A* is the Hamaker constant (Equation (3)):

(5) $$G_{LVW(cell-cell)}=\;-\frac{Ar_{1}r_{2}}{6d\left(r_{1}+r_{2}\right)}$$

The Lewis acid-base interaction, or the hydrophobic/hydrophilic interaction between a spherical particle and a semi-infinite plate ($G_{AB}$) is calculated as [144]:

(6) $$G_{AB}=2\pi r\lambda {∆G}_{adhesion}^{AB}\exp\left(\frac{d_{0}-d}{\lambda }\right)$$

where *λ* is the correlation length, which is equal to 6 × 10^−10^ m for an attractive interaction (hydrophilic) and ranges between 1 × 10^−10^ and 2 × 10^−10^ m for a repulsive interaction (hydrophobic) [145].Where *λ* is the correlation length, which is equal to 6 × 10^−10^ m for an attractive interaction (hydrophilic) and ranges between 1 × 10^−10^ and 2 × 10^−10^ m for a repulsive interaction (hydrophobic) [145].

In the case of two interacting spheres with radii *r*_1_ and *r*_2_, the Lewis acid-base interaction is calculated using the following equation:

(7) $$G_{AB}=2\pi \frac{r_{1}r_{2}}{r_{1}+r_{2}}\lambda {∆G}_{AB}\exp\left(\frac{d_{0}-d}{\lambda }\right)$$

The change in free energy component due to the Lewis acid-base interactions (${∆G}_{adhesion}^{AB}$) is calculated as [142]:

(8) $${∆G}_{adhesion}^{AB}=2\left[\left(\sqrt{\gamma_{c}^{+}}-\sqrt{\gamma_{cl}^{+}}\right)\left(\sqrt{\gamma_{c}^{-}}-\sqrt{\gamma_{cl}^{-}}\right)-\left(\sqrt{\gamma_{c}^{+}}-\sqrt{\gamma_{L}^{+}}\right)\left(\sqrt{\gamma_{c}^{-}}-\sqrt{\gamma_{L}^{-}}\right)-(\sqrt{\gamma_{cl}^{+}}-\sqrt{\gamma_{L}^{+}})(\sqrt{\gamma_{cl}^{-}}-\sqrt{\gamma_{L}^{-}})\right]$$

where $\gamma^{-}$ and $\gamma^{+}$ denote the electron donor (basic component) and acceptor (acid component), respectively, and the subscripts *c*, *cl*, and *L* denote the microalgal cell, the clay particle, and the water medium, respectively.

Studying acid-base interactions through surface energetics analysis is crucial for understanding the physicochemical interactions between microalgal cells and clay nanoparticles using the XDLVO approach [146,147,148]. This theory has been applied by several authors to examine how clay characteristics, surface charge, and hydrophilicity influence the flocculation of harmful algae. Specifically, the XDLVO framework was used to evaluate the efficiency of various clays—montmorillonite-K10, montmorillonite-KSF, kaolinite, and loess—in flocculating two microalgal species: *Microcystis* sp. (freshwater) and *Prorocentrum minimum* (marine) [149]. Both species are well-known HAB formers. The findings indicate that acid-base interactions are the primary flocculation mechanism for *P. minimum* [149], whereas electrostatic interactions dominate in the case of *Microcystis* sp. in freshwater systems.

The electrostatic component of free energy ($G_{EL}$) between a spherical particle and a semi-infinite plate is calculated as [142]:

(9) $$G_{EL}=\pi \varepsilon r\left(\psi_{c}^{2}+\psi_{s}^{2}\right)\frac{2\psi_{c}\psi_{s}}{\psi_{c}^{2}+\psi_{s}^{2}}\ln\left(\frac{1+e^{-2kd}}{1-e^{-2kd}}\right)+\ln\left(1-e^{-2kd}\right)$$

where ε is the water permittivity (ε = 6.88 × 10^−10^ F.m^−1^), r is the microalgal cell radius (m), $\psi_{c}$ and $\psi_{s}$(V) are the surface potentials of the microalgal cell and the semi-infinite plate, respectively, with the subscripts c and s referring to the microalgal cell and the semi-infinite plate, respectively, d is the separation distance (m), and 1/*k* is the double layer thickness (m) given by [142]:

(10) $$k={\left(\frac{e^{2}}{\varepsilon kT}\sum_{i}z_{i}^{2}n_{i}\right)}^{1/2}$$

where *e* is the electron charge (*e* = 1.6022 × 10^−19^ C), *k* is the Boltzmann constant, *z_i_* is the charge number for ions, and *n_i_* is the ions concentration of the ion in the solution. Nabweteme et al. [149] have reported values for the layer thickness κ^−1^ of 8.963 nm for the freshwater and 0.371 nm for the artificial seawater [149]. The surface potentials of the microalgal cell and the substrate (ψ) are calculated using:

(11) $$\psi =\;\zeta \left(1+\frac{v}{r}\right)e^{kv}$$

where *ζ* is the zeta potential, v is the thickness of the hydration layer (m) of the microalgal cells and *r* is the radius of the microalgal cell (m). The thickness of the hydration layer is inversely proportional with the ionic strength of the medium and its value ranges from $3\times 10^{-11}\;to\;5\times 10^{-11}\;m$ [150,151]. Similarly, the electrostatic component of free energy between two interacting spheres of radii *r*_1_ and *r*_2_ ($G_{EL}$) is calculated as follows:

(12) $$G_{EL\;(cell-cell)}=\frac{\pi \epsilon r_{1}r_{2}\left(\psi_{c}^{2}+\psi_{s}^{2}\right)}{\left(r_{1}+r_{2}\right)}\frac{2\psi_{c}\psi_{s}}{\psi_{c}^{2}+\psi_{s}^{2}}\ln\left(\frac{1+e^{-2kd}}{1-e^{-2kd}}\right)+\ln\left(1-e^{-2kd}\right)$$

## 5. Improving the Efficiency of Nano Clays in Controlling *Alexandrium* spp.

Improving the efficiency of clay-based treatments is essential for advancing their cost-effectiveness and expanding their practical utility. By reducing the amount of clay required, treatments become more economically feasible, especially for large scale areas or for locations where the benefits, such as environmental protection or the preservation of social and cultural values, are less easily measured in economic terms. Additionally, using less clay helps reduce unintended impacts on non-target species, making the approach more environmentally sustainable.

Natural clay has very low flocculation and coagulation efficiencies, which can result in significant clay deposition to achieve effective removal in field applications [76,80,124]. This can lead to large deposition loads on sediments, which can cause deleterious effects on the benthic life of benthic marine life and broader marine ecosystem organisms and the marine ecosystem [124,152]. For example, in Korea, the clay dosage used to control *Margalefidinium polykrikoides* was reported to be 384 t km^−2^ [59]. This resulted in high dosage with low removal efficiency, large deposition loads on sediment, higher cost, and greater logistical challenges [152]. Consequently, much research has conducted to reduce the amount of clay required for treatment and increase the removal efficiency [110,113,118,125].

## 6. Challenges and Opportunities

### 6.1. Environmental Implications and Risk Assessment

To date, the application of modified nano clays has been limited due to several key factors. Typically, the costs associated with the production and application of modified nano clay can be prohibitive, especially for large-scale application [63,80,81,101]. These high costs present limitations, especially when using the treatments on a large scale, as the cost of clay may account for only approximately 1% of the total clay treatment cost while vessel and clay dispenser use account for over 70% of the cost [76]. In addition, the use of modified nano clay for the treatment of *Alexandrium* spp. may impact product quality and quantity and have unintended economic effects on commercial and recreational fisheries and aquaculture farms [76].

The use of modified nano clays to treat *Alexandrium* spp. may also cause harm to both benthic and pelagic marine environments [63,80,101]. Modified nano clay may attach to non-target aquatic organisms during flocculation and settlement flocs may negatively affect benthic flora and fauna [56,104,153]. Flocculation and settlement can also reduce the feeding activity of visual predators, such as fish, due to increased turbidity in the aquatic environment [56]. Table 4 summarises the impacts of modified nano clays on various organisms.

Additionally, the technology of using MC has also advanced to the point of using organic modifiers/flocculants [162], which has gained more recognition in recent years due to the nature of it being low in as they can reduce toxicity and, are often biodegradable and thus improving the ecological acceptability of this management approach. However, there is still a substantial amount of research that is required before widespread use of organic surfactants are rolled out on a large scale [40]. Validation of the use of nano clays modified with organic flocculants should be a major focus for researchers and environmental managers, and yet the problem of treating and controlling HAB’s in general, and *Alexandrium* blooms, in particular, is pressing.

While modified nano clays show promise for treating *Alexandrium* spp. blooms, their field effectiveness remains to be fully evaluated, particularly concerning the deposition and resuspension of clay/algae flocs and potential impacts on local aquatic systems, including shellfisheries and aquaculture farms [63,80,81,101]. Comprehensive ecotoxicological assessments at the mesocosm scale are needed to determine effects on ecosystems, non-target species, and benthic habitats, ensuring minimal environmental harm.

### 6.2. Reducing HAB Toxicity

Modified clays have proven effective in significantly reducing toxicity of HABs. The mechanisms behind this reduction include adsorption of extracellular toxins, inhibition of toxin biosynthesis, alteration of toxin composition, and simple sedimentation of cell-bound toxins [163]. In one study, PAC-MC treatment during the removal of *A. tamarense* was found to convert the highly toxic GTX1/4 into a less toxic decarbamoyl derivative dcGTX2 and dcGTX3 via catalytic conversion [112] resulting in over 80% reduction of *A*. *tamarense* toxicity. In some cases, toxin removal of *A. pacificum* reached up to 90% [128]. Additionally, clay concentrations between 0.01 g L^−1^ have been shown to inhibit the production of domoic acid in *Pseudo-nitzschia pungens* and *Pseudo-nitzschia multiseries* [164].

Despite these benefits, concerns remain regarding the fate of toxins within sedimented flocs. Some studies suggest that benthic filter feeders, such as shellfish, may accumulate toxins by ingesting deposited algal cells, particularly *Karenia brevis* [165]. However, other research indicates that MC treatment disrupts algal cells and dilutes toxins in the water, thereby reducing the concentration of toxins available for uptake by shellfish compared to direct ingestion during active blooms [154]. Toxin uptake is influenced by multiple factors, including species-specific traits, water turbulence or disturbances, and feeding behaviours [56,101]. Ultimately, the mechanisms underlying toxicity reduction must be explored comprehensively.

Modified clays have shown promise in reducing the toxicity of saxitoxins through adsorption. The long-term fate of these toxins remains uncertain, as they can become bound within clay flocs and persist in sediments, potentially re-entering the food chain [102]. Although treatment with PAC-MC has demonstrated a reduction in toxin concentration, this suggests that a significant portion of the toxins may accumulate in sediments following flocculation [112,154]. The potential for remobilisation of these bound toxins under anoxic conditions or physical disturbances, such as sediment resuspension has not been fully explored. Further investigations are needed to determine how long these toxins remain bound, the environmental conditions that may trigger their release, and whether they become bioavailable to benthic and pelagic organisms. Understanding these dynamics is essential for assessing the long-term ecological implications of modified clay (MC) applications.

### 6.3. Stability

Nano clays are capable of undergoing biodegradation over time due to exposure to ultraviolet (UV) radiation, chemical interactions in aquatic environments, and microbial activity [166]. In experimental studies, focusing on physical behaviour of natural clays with or without polyaluminum hydroxychloride PACl, clay flocs were observed to resuspend after approximately three hours of settling, particularly under increased flow velocities [56,167]. However, over longer durations, the flocs exhibited enhanced resistance to resuspension. This increased stability was attributed to dewatering and compaction processes, which reduce the likelihood of erosion. Interestingly, the long-term stability of flocs was significantly improved when nano clay was co-applied with PACl. While PACl enhanced flocculation efficiency, the structural integrity of flocs was more robust when clay was used in conjunction with coagulants, compared to unmodified clay alone [56,168].

### 6.4. Cost/Sustainability

Over the past decade, Western Australia (WA) has experienced a concerning rise in the detection of *Alexandrium* spp. and subsequent blooms. Notably, the Swan Canning Estuary, a cherished attraction in WA, encountered two significant *Alexandrium* bloom events in 2019 and 2020 [35]. The implications of these blooms on recreational fishers, and the overall ecosystem [5,8,169]. Notably, the Swan Canning Estuary, a cherished attraction in WA, encountered two significant *Alexandrium* bloom events in 2019 and 2020 [35]. The implications of these blooms on recreational fishers, and the overall ecosystem [5,8,169] make it imperative to carefully consider a cost-effective treatment of *Alexandrium* blooms. Modified nano clays have emerged as one of the most promising strategies for harmful algal bloom mitigation [80]. Previous laboratory studies have demonstrated their effectiveness in flocculating cells of *Alexandrium tamarense* [112] and *Alexandrium pacificum* [37]. Notably, a removal efficiency of up to 90% was achieved for vegetative cells using 0.25 g L^−1^ of modified clay in a 50 mL algal culture with an approximate cell density of 3.1 × 10^7^ cells L^−1^ [86]. The modified clay slurry used in these experiments consisted of one-part polyaluminum chloride (PACl) to five parts kaolin, mixed in ultrapure water [37,112]. For large-scale applications, the use of seawater as the mixing medium, rather than ultrapure water, would offer a more cost-effective and practical approach, thereby improving the overall economics of slurry preparation [13].

An important consideration in the application of modified nano clays is the cost of the modifier, in this case PACl. While PACl enhances removal efficiency and reduces the amount of kaolinite required, thereby lowering the overall clay load in marine ecosystems [111], its cost may exceed that of kaolinite. Thus, although PACl contributes to improved treatment performance and potential cost savings in clay usage, it introduces an additional expense.

Here we incorporated cost estimates based on the findings of previous studies [37,112], comparing the relative expenses associated with kaolinite and PAC in mitigation strategies. These can be coupled with the use of literature data regarding removal efficiencies, et., a For, example, a removal efficiency of 90% was reported at a kaolinite concentration of 0.25 g L^−1^ (equivalent to ~350.2 kg ha^−1^) [112], although this level of efficacy is limited to vegetative cells of *Alexandrium* species at densities up to 3.1 × 10^7^ cells L^−1^. For higher cell densities, doubling the concentration of both kaolinite and PAC to 0.5 g L^−1^ (around 700.4 kg ha^−1^) is recommended to maintain treatment effectiveness.

Using 2022 Australia market prices, of USD $0.16 per kg (https://wakaolin.com.au/ accessed: 25 January 2025) and USD $0.29 per kg (https://www.waterpurifyingchemicals.com/, accessed: 25 January 2025) for kaolinte, and industrial-grade PACl, respectively. The estimated material cost for treating a 1-hectare bloom at a PACl:kaolinite ratio of 5:1 would be approximately USD $94. In the case of the recent WA *Alexandrium* blooms [35], which spanned 1900 hectares, the total material cost would be around $178,000. However, this cost could be significantly reduced through early detection and intervention, allowing treatment prior to the bloom reaching its full extent.

Scenario-based cost-benefit analysis should be conducted prior to the application of modified clay (MC) treatments. This way, waterbody and asset managers will be pre-informed of approximate costs and operational requirements, promoting a more rapid and decisive response. Most economic assessments of HABs rely on a market value approach, which estimates losses based on reduced outputs in commercial sectors such as aquaculture and shellfisheries [46,170]. However, this method does not account for non-market impacts, such as those associated with recreational fishing that operates outside formal economic systems [49], which can make it difficult to capture the full scope of the losses incurred. Consequently, the true scope of losses, particularly those affecting ecosystem services, public health, and cultural or recreational values, may be underestimated in traditional economic evaluations.

Evaluating the feasibility of modified clay (MC) for large-scale harmful algal bloom mitigation requires consideration of both economic and environmental costs, particularly the carbon footprint associated with MC production. Carbon emissions vary depending on the type of modification, raw materials, and processing methods. For example, a recently proposed method for preparing modified clay [171] involves a multi-step activation and modification process, with calcination identified as the most carbon-intensive stage (see Table 5). In comparison, the use of unmodified clay or co-applied clay (e.g., with PACl) avoids energy-intensive modification processes, resulting in a relatively lower carbon footprint—at least during the production phase. While transitioning from fossil fuels to renewable energy sources such as solar, wind, and hydropower can significantly reduce carbon emissions, it is important to recognize that these alternatives also carry environmental costs. Solar energy relies on batteries for energy storage, and disposal of these batteries poses an environmental challenge [172]. Wind turbines, on the other hand, have been associated with bird fatalities and are considered by some as visual pollutants, while hydropower can disrupt aquatic ecosystems [173,174]. Given these trade-offs, the authors recommend careful consideration of the post-production costs of renewable energy sources when assessing the economics of HAB treatments with modified nano clays. The following comparison is limited to a production-based life cycle assessment (LCA) and does not account for cradle-to-cradle impacts such as transportation, application, and post-treatment environmental effects.

Considering these factors, co-applied clays present a compelling solution for the cost-effective and efficient treatment of *Alexandrium* blooms [13]. Early intervention using suitable concentrations of modified nano clay can help mitigate the impact on waterways and minimize the economic burden on affected regions. The potential benefits of this approach highlight the importance of further research and practical application to better manage and safeguard aquatic ecosystems from the harmful effects of *Alexandrium* blooms. Modified nano clay stands among the limited physio-chemical treatments implemented on a large scale in the environment [59,62,81].

## 7. Conclusions and Future Directions

This review set out to explore management strategies for prevention and control toxic *Alexandrium* spp. blooms. We showed that, the effective management requires a multifaceted and sustainable approach that minimises environmental impacts while protecting public health and economic interests. Policymakers and water management authorities should consider a combination of strategies such as routine *Alexandrium* surveillance, regular risk assessments, and using clay treatment as an emergency treatment measure in affected waterways. These measures can enhance responsiveness and reduce the impacts of bloom events. In addition, management authorities should consider incorporating rapid diagnostic tools (eg qPCR) and seafood testing to ensure timely intervention and reduce risk to consumers.

Overall, due to the complexities associated with using modified nano clays, management of *Alexandrium* spp. should not rely solely on their application. Instead, modified nano clay treatment should be integrated into a broader, holistic process that includes monitoring, prevention, and other management approaches to reduce dependence on reactive treatment methods. Consequently, the use of modified nano clays should be considered an emergency response rather than a routine management tool. It is also essential to identify the optimal conditions for their application to enhance removal efficiency and to establish a clear treatment plan for responding to bloom emergencies. Given our current level of knowledge, further work is needed to better define the preventative potential of modified nano clays and to assess their post-production impacts.

Currently, there is limited research specifically addressing the carbon footprint of modified clays (MCs) used in harmful algal bloom (HAB) treatment. The few existing studies emphasize the importance of evaluating the full life cycle of these materials to ensure that potential environmental trade-offs are thoroughly understood, strongly suggesting that this information is not yet readily available. Therefore, further research is needed to develop comprehensive life cycle assessments (LCAs) that encompass all stages of MC production and application, enabling more informed and sustainable decision-making in environmental management.

Public education programs and initiatives should also be encouraged to minimise exposure risks during bloom events, especially among communities that value the affected waterways for their cultural significance and recreational use. Finally, consistent monitoring of water bodies, coupled with the implementation of clear and actionable guidelines, will strengthen the resilience and effectiveness of management strategies against *Alexandrium* spp. blooms.

## Figures and Tables

**Table 2:** Treatment of various HAB species using modified nano clays.

HAB Species	Modified Clay Treatment	Conc Clay (g L^−1^)	Environmental Conditions Tested	Removal Efficiency (RE) (%)	Findings	Ref.
*Aureococcus anophagefferens*	Aluminium sulfate-Modified clay (AS-MC)	0.1–2.0	pH: 3–11; DW vs. SW; pH: 7–11; SW; Temp: 15–25 °C	95—5% (pH 3–7, DW) ~70% (pH 7–11, SW)	DW: 95%–5%; SW: 95%–30% (pH 3–7); Improves to ~70% at pH 11	[118]
					SW: 30% at pH 7 increases to ~70% at pH 11	
	Aluminium chloride (AC-MC)	0.1–2.0	pH: 3–11; DW vs. SW; pH: 7–11; SW; Temp: 15–25 °C	95—22% (pH 3–7, DW) ~70% (pH 7–11, SW)	DW: 95%–22%; SW: 95%–35%; up to ~70% at pH 11;	
					SW: 30% at pH 7 increases to ~70% at pH 11	
	Poly aluminium chloride (PAC-MC)	0.1–2.0	pH: 3–11; DW vs. SW; pH: 5–11; SW; Temp: 15–25 °C	95—20% (pH 3–7, DW) ~75% (pH 7–11, SW)	DW: ~90% at pH 3; SW: 20–60% (pH 3–5); ~75% at pH > 7	
					SW: 20% at pH 5 increases ~75% at pH 11	
*Phaeocystis globosa*	PAC-MC	0.5–1.0	pH: 3 to 11; temp: 10–40 °C	26–44.6% (sulphate removed) 24.5% (sulphate restored) ~75% at pH 11	Salinity improves when sulfate is removed; increase in pH increases RE.	[104]
*Skelotonema costatum*	PAC-MC	0.025–2.00	Turbidity, pH stability,	98.79% at 1 g L^−1^; >97% at 0.25 g L^−1^; 98.98% with sediment; 97.81% without sediment	0.25 g L^−1^ preferred due to high RE minimal turbidity; sediment enhances removal. pH > 7.77 supported effective coagulation prevented re-blooming	[102]
*Scrippsiella trochoidea* cysts	Kaolin, and polymeric aluminium chloride PACl	0.1, 0.5 & 1.0	Removal efficiency. final cyst formation; germination rate.	69.1%—0.1 g L^−1^; 94.3%—0.5 g L^−1^; 97.7%—1.0 g L^−1^	Cyst formation increased with clay concentration (17.9%, 22% & 24.6%); germination rate decreased with increase in clay concentration (71.3%, 47.5% & 53.3%).	[119]
*Prymnesium parvum*	Wet Bentonite + PAC	0.05 & 0.5	pH: 8; temp: 20 °C; cell densities: (1 × 10^6^ & 1 × 10^8^ cells L^−1^).	64%—0.05 g L^−1^; 77%—0.5 g L^−1^	Highest removal 0.5 g L^−1^ & at lower cell density temperature, pH and salinity kept constant.	[120]
*Gymnodinium breve*	Florida hosphatic clay (IMC-P2 + PAC)	0.25	pH: 6.98 Temp: 20 °C	~90%	PAC enhanced removal	[107]
*Aureococcus anophagefferens*	Kaolinite acid treated clay (H-DP)	4.0	pH: 4.86 Temp: 20 °C	~85%	Required mixing for effective removal	
*Margalefidinium polykrikoides*	Sophorolipid-yellow-modified clay (SMC)	0.01 & 0.02	Temp: 20 °C	0.01 g L^−1^—80% 0.02 g L^−1^—90%		[56]
*Margalefidinium polykrikoides*	SMC	0.005 (Sophorolipid) + 1.0 (yellow clay)	pH: 3.5 Temp: 25 °C	95%	95% removal efficiency within 30 min, outperforming yellow clay alone (10 g L^−1^)	[121]
*Chattonella marina*	Ethylene bis (dodecyl dimethyl ammonium bromide) (EDAB)	0.003	Temp: 22 °C	3.0 mg/L 100%	Doses depend at higher removal efficiency and higher dose	[122]
*Microcystis aeruginosa*	Hexadecyltrimethyl ammonium bromide (CTAB)	0.02 (clay-lake sediment) + 0.8 CTAB	Temp: 22 °C	98.92%	CTAB concentration 0.1 to 1 g L^−1^ had RE of >90%	[123]

**Table 3:** Treatment of various species of *Alexandrium* spp. using modified nano clays.

HAB Species	Modified Clay Treatment	Conc Clay (g L^−1^)	Environmental Conditions Tested	Removal Efficiencies (RE) (%)	Findings	Ref.
*Alexandrium tamrense*	Kaolinite clay + Polyaluminum chloride (K-PAC)	0.25 g L^−1^	Temp: 20 ± 1 °C Cell densities: (3.1 × 10^7^ cells L^−1^)	>90%	RE increased when concentration of K-PAC increased (2.0 g L^−1^—99.35%); detoxification of PSTs Nutrient removal	[112]
	Montmorillonite intercalated with palmityl sulfobetaine	0.02 g L^−1^	pH: 8 Temp: 22 ± 1 °C Cell densities: (3.1 × 10^6^ cells L^−1^)	~70%	Removal increased with sulfo betaine content in clay.	[42]
*Alexandrium pacificum*	(K-PAC)	0.2, 0.4, 0.6 & 0.8 g L^−1^	Temp: 20 ± 1 °C pH: 8.9 Cell densities: (1.0 × 10^4^ cells L^−1^)	1 g L^−1^—~99% 0.6 g L^−1^—~90% 0.2 g L^−1^—~75%	RE increased with increase in concentration of K-PAC	[37]
	Kaolin + potassium peroxymonosulfate (PMPS-MC)	0.005 & 0.01 g L^−1^	Temp: 20 ± 1 °C Cell densities: (2.3 × 10^6^ cells L^−1^)	>95%	RE achieved within 3 h; increased when pH was >8	[128,129]
	(PMPS-MC) (Toxin removal experiment)	0.005 & 0.01 g L^−1^	Temp: 20 ± 1 °C Cell densities: (2.3 × 10^6^ cells L^−1^)	>93%	29–46% toxin reduction via transformation (i.e., GTX1&4 to GTX2&3 and C1&C2) in 15 min; 46–50% toxicity reduction compared to control	
*Alexandrium minutum*	Kaolinite + Polyaluminum chloride (KPAC)	0.05, 0.1, 025 & 0.3 g L^−1^	Salinity: ~32 psu Temp: 20 ± 1 °C pH: 7 & 8 Cell densities: (1.0 × 10^7^ & 2.0 × 10^7^ cells L^−1^)	100%	Best RE 0.1 g L^−1^ with no significant difference in RE compared to other concentrations; pH had no significant effect. KPAC prepared with seawater	[13]
HAB species	Modified clay treatment	Conc clay (g L^−1^)	Environmental conditions tested	Resting cyst formation and germination rate	Findings	Ref.
*Alexandrium pacificum* cysts	Kaolinite clay + Polyaluminum chloride (K-PAC)	0.2, 0.4, 0.6 & 0.8 g L^−1^	Temp: 20 ± 1 °C pH: 8.9 Cell densites: (1.0 × 10^4^ cells L^−1^)	formation rate Control—29.7% 1 g L^−1^—~12% 0.6 g L^−1^—~15.5% 0.2 g L^−1^—~30%	Higher MC reduced resting cyst formation and germination; MC concentrations > 0.4 g/L reduced “seed” cysts for future blooms	[37]
				germination rate Control—68.0% 1 g L^−1^—~26.5% 0.6 g L^−1^—~171.4% 0.2 g L^−1^—~68.6%		

**Table 4:** Summary of ecotoxicological studies assessing effects of modified nanoclay on marine and estuarine organisms.

Species Tested	Clay Type & Concentration	Study	Exposure/Conditions	Findings	Ref.
*Argopecten irradians* *(Bay scallop)*	Modified clay 0.1 & 0.5 g L^−1^	Laboratory	16-day exposure to *Alexandrium tamarense* bloom	MC treatment: toxin accumulation in scallop tissues was greatly reduced: ~13.5% of initial toxin incorporated at 0.1 g L^−1^, and almost none at 0.5 g L^−1^. Toxins in sediments were rapidly detoxified, falling below detection within 4 days, whereas toxins persisted in scallop tissues for ~16 day	[154]
*Ampelisca abdita*, *Leptocheirus plumulosus* (Infaunal amphipods)	Phosphatic clay + polyaluminum hydroxy chloride (PAHC) 0.0005, 0.005 & 0.05 g L^−1^	Laboratory	Acute and chronic toxicity tests with *Karenia brevis* and phosphatic clay + PAC	Phosphatic clay + PAC; mostly non-toxic; *K. brevis* moderately toxic to *L. plumulosus*	
*Cyprinodon variegatus* (Larval sheepshead minnows)				*K. brevis* alone: highly toxic; Phosphatic clay + PAHC; did not consistently reduce toxicity; Phosphatic clay alone; mostly non-toxic	[155]
*Palaemonetes pugio* (Embryos of grass shrimp)				Phosphatic clay + PAC mostly non-toxic	
*Brachionus plicatilis* (Rotifer)	PAC-modified clay 0.1–1.0 g L^−1^	Simulated laboratory bloom	2 h and 2 d exposures during *K. mikimotoi* bloom	0.1 g L^−1^: minimal effect on population and reproduction. higher concentrations (0.5–1.0 g L^−1^) not tested on rotifers specifically	[156]
*Mytilus edulis* (Blue mussels)			Sedimentation to benthos during *K. mikimotoi* bloom	0.1 g L^−1^: minimal impact; 0.5–1.0 g L^−1^: gill/digestive gland damage, reduced filtering rate, enzymes, condition index, and increased mortality	
*Callinectes sapidus* (Adult blue crabs)	PAC-modified clay (Modified Clay II) 0.5 g L^−1^	Laboratory	8-day exposure to clay alone, *K. brevis*, or clay + *K. brevis*	PAC-modified clay alone: no effect; PAC-modified clay reduced *K. brevis* concentration by 95%; no significant impact on mortality or behavioral reflexes	[157]
*Callinectes sapidus* (Adult blue crabs)	PAC-modified clay (Modified Clay II) 0.2 g L^−1^	Simulated bloom in 1400 L mesocosm	72-h exposure with *K. brevis* bloom-level density (1 × 10^6^ cells/L)	RE of 57% of *K. brevis* cells after 8 h and 95% after 48 h; No significant lethal or sublethal effects on blue crabs, sea urchins, or hard clams at 72 h; Suggests MC II at 0.2 g/L is environmentally safe for these benthic species under short-term exposure	[158]
*Lytechinus variegatus* (Sea urchin)					
*Mercenaria campechiensis* (Hard clam)					
*Haliotis discus hannai* (Abalone)	Modified clay	Laboratory and field experiments	Short-term (1–15 days) and recovery period (16–30 days) in lab; typical aquaculture field conditions	Survival: No significant effect at tested concentrations Decrease in shell length and weight day 1–15 rapid recovery day 16–30	[159]
*Mercenaria mercenaria* (Juvenile hard clams)	Phosphatic clay	Large flume (simulated field)	2-week exposure, low-flow (sedimentation) vs. high-flow (suspension) with nontoxic algae	Low-flow: minor or no growth inhibition; High-flow: strong growth reduction (~90%); no mortality in either case	[160]
*Scophthalmus maximus* (Turbot embryos)	PAC-modified clay 0–1.7 g L^−1^	Large flume (simulated field)	24–48 h exposure	LC50: 1.70 g L^−1^ (24 h), 1.65 g L^−1^ (48 h); Safe concentration: 0.47 g L^−1^; Hatchability not affected at ≤0.5 g L^−1^; deformities increased above 0.5 g L^−1^; growth and yolk absorption peaked at g L^−1^, then decreased	[161]

**Table 5:** Proposed new method for preparing modified clay to control HABs [171].

Modified Clay Process	Procedure	Relative Carbon Impact
1. Calcination	750 °C for 2 h	High (most energy intensive)
2. Acid etching	Sitrred at stirred for 40 minuted at a fixed stirring rate of 400 r/min at 93 °C.	Medium
3. Alkali neutralization	Addition of Sodium hydroxide (NaOH) to adjust pH	Low
4. Raw clay supplementation	Addition of raw clay to ratio 2:1(addition of weight)	Low
5. Aging	at 500 rpm at 70 °C for 3 h	Low-medium
6. Drying	70 °C	Low-medium

## Data Availability

No new data were created or analyzed in this study.

## Abbreviations

The following abbreviations are used in this manuscript:

MC	Modified clay
PACl	polyaluminum hydroxy chloride
PMPS-MC	potassium peroxymonosulfate modified clay
RE	Removal efficiency
WA	Western Australia
PAC	Polyaluminum chloride
HAB	Harmful algal bloom
PST	Paralytic shellfish toxin
NaCl	Sodium chloride
NaOH	Sodium hydroxide
LCA	Life cycle assessments
